# Proactive prevention: Act now to disrupt the impending non-communicable disease crisis in low-burden populations

**DOI:** 10.1371/journal.pone.0243004

**Published:** 2020-12-01

**Authors:** Benson Njuguna, Sara L. Fletcher, Constantine Akwanalo, Kwaku Poku Asante, Ana Baumann, Angela Brown, Victor G. Davila-Roman, Julia Dickhaus, Meredith Fort, Juliet Iwelunmor, Vilma Irazola, Sailesh Mohan, Vincent Mutabazi, Brad Newsome, Olugbenga Ogedegbe, Sonak D. Pastakia, Emmanuel K. Peprah, Jacob Plange-Rhule, Gregory Roth, Archana Shrestha, David A. Watkins, Rajesh Vedanthan

**Affiliations:** 1 Department of Pharmacy, Moi Teaching & Referral Hospital, Eldoret, Kenya; 2 Department of Cardiology, Moi Teaching & Referral Hospital, Eldoret, Kenya; 3 Oregon State University College of Pharmacy, Corvallis, OR, United States of America; 4 Kintampo Health Research Centre, Kintampo, Brong Ahafo Region, Ghana; 5 Brown School of Social Work, Washington University in St. Louis, St. Louis, MO, United States of America; 6 Cardiovascular Division, Washington University School of Medicine, St. Louis, MO, United States of America; 7 Department of Population Health, New York University Grossman School of Medicine, New York, NY, United States of America; 8 Colorado School of Public Health, Aurora, CO, United States of America; 9 Department of Behavioral Science and Health Education, College for Public Health and Social Justice, Saint Louis University, St. Louis, MO, United States of America; 10 Department of Research in Chronic Diseases, Institute for Clinical Effectiveness and Health Policy (IECS), Buenos Aires, Argentina; 11 Public Health Foundation of India (PHFI), Gurgaon, Haryana, India; 12 Centre for Chronic Disease Control (CCDC), Gurgaon, Haryana, India; 13 Regional Alliance for Sustainable Development–Rwanda, Kigali, Rwanda; 14 Center for Translation Research and Implementation Science, National Heart, Lung, and Blood Institute, National Institutes of Health, Bethesda, MD, United States of America; 15 Purdue University College of Pharmacy, Center for Health Equity and Innovation, West Lafayette, IN, United States of America; 16 Department of Pharmacology, Moi University School of Medicine, Eldoret, Kenya; 17 Indiana Institute for Global Health, Indianapolis, IN, United States of America; 18 Department of Social and Behavioral Sciences, Global Health Program, NYU School of Global Public Health, New York, NY, United States of America; 19 Ghana College of Physicians and Surgeons, Accra, Ghana; 20 Division of Cardiology, University of Washington, Seattle, WA, United States of America; 21 Department of Public Health, Kathmandu University School of Medical Sciences, Dhulikhel, Kavrepalanchwok, Nepal; 22 Department of Global Health, University of Washington, Seattle, WA, United States of America; Federation University Australia, AUSTRALIA

## Abstract

Non-communicable disease (NCD) prevention efforts have traditionally targeted high-risk and high-burden populations. We propose an alteration in prevention efforts to also include emphasis and focus on low-risk populations, predominantly younger individuals and low-prevalence populations. We refer to this approach as “proactive prevention.” This emphasis is based on the priority to put in place policies, programs, and infrastructure that can disrupt the epidemiological transition to develop NCDs among these groups, thereby averting future NCD crises. Proactive prevention strategies can be classified, and their implementation prioritized, based on a 2-dimensional assessment: impact and feasibility. Thus, potential interventions can be categorized into a 2-by-2 matrix: high impact/high feasibility, high impact/low feasibility, low impact/high feasibility, and low impact/low feasibility. We propose that high impact/high feasibility interventions are ready to be implemented (act), while high impact/low feasibility interventions require efforts to foster buy-in first. Low impact/high feasibility interventions need to be changed to improve their impact while low impact/low feasibility might be best re-designed in the context of limited resources. Using this framework, policy makers, public health experts, and other stakeholders can more effectively prioritize and leverage limited resources in an effort to slow or prevent the evolving global NCD crisis.

## Introduction

The majority of global non-communicable disease (NCD) burden is attributable to modifiable behavioral and environmental risk factors: tobacco use, unhealthy diet, physical inactivity, harmful alcohol use, and air pollution [1]. These risk factors have been highlighted as both high priority, and cost-effective targets for NCD prevention and control [2]. In many regions of the world, and in many populations, prevention efforts such as healthy eating, increasing physical activity, and smoking cessation focus on individuals who are already at high risk (e.g. due to the presence of metabolic risk factors) and populations with high prevalence of NCD, and are therefore largely “reactive” [2, 3]. These efforts reduce NCD prevalence, morbidity, and mortality, and as such need to continue.

However, there is an important gap. If the “reactive prevention” approach alone is implemented around the world, the global NCD community will likely soon be confronting a situation of “too little, too late.” To complement these efforts, there is therefore a concomitant need to target individuals at low risk and populations with relatively lower burden at the current critical time period. We call this approach “**proactive prevention**,” which refers to implementation of public health policy and other interventions that create environments and facilitate behaviors to disrupt the epidemiological transition and maintain the lower burden of low-risk individuals and low-NCD prevalence populations.

The most compelling reason to initiate proactive prevention is to slow or eliminate the development of a high disease burden at the population level. Even in countries with already high NCD burden, segments of the population, particularly children and adolescents, are at low risk or have not yet been afflicted [4]. Early health promotion efforts could delay or wholly prevent progression to overt NCDs over the course of their lifetime [4, 5], thus reducing premature death and disability. The critical window to pursue proactive prevention will soon no longer be present and the time to act is now!

In this paper, we first define and characterize low-burden and low-risk populations who have the highest potential benefit from proactive prevention strategies. We then propose a novel framework using concepts of impact and feasibility to stratify and prioritize potential proactive prevention interventions, highlighting evidence-based examples. Finally, we suggest a way forward for proactive prevention strategies to be realized. Our overall framework was initially developed by three of co-authors (RV, BN, SF), then modified in an iterative fashion in consultation and discussion with all co-authors. The final framework and approach was approved by consensus by all co-authors.

## Target populations for proactive prevention

**Fig 1:**
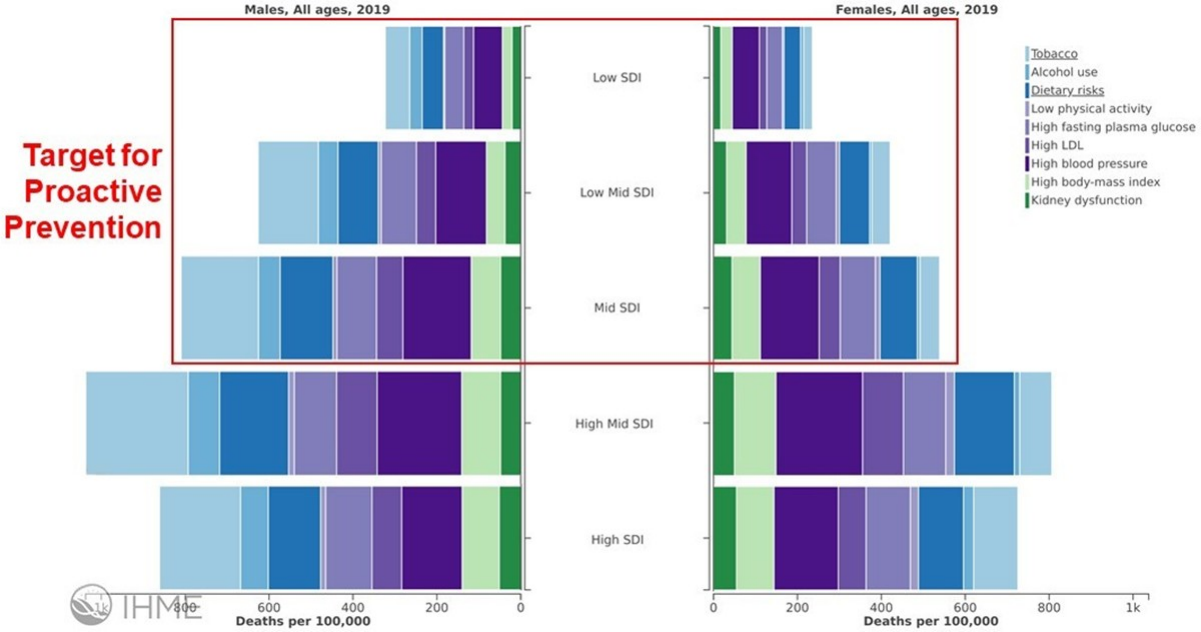
Deaths per 100,000 attributed to NCD risk factors, stratified by socio-demographic index (SDI) [6]. The potential for proactive prevention in relatively lower-burden populations is highlighted.

**Fig 2:**
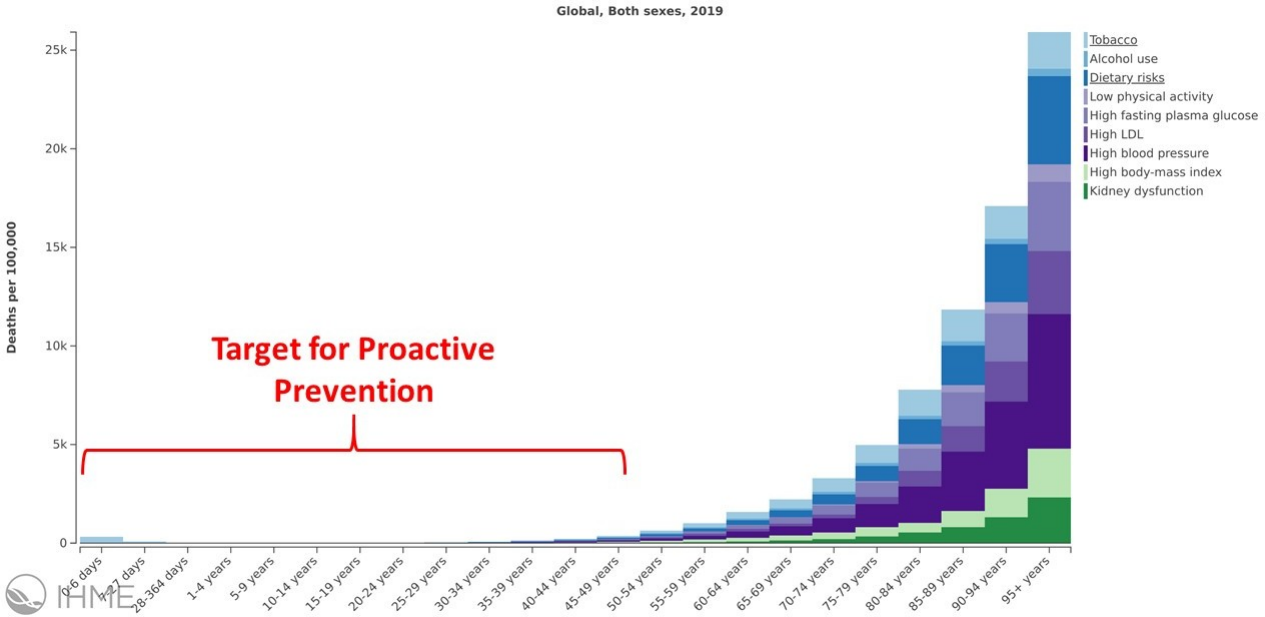
Global death rate attributed to metabolic risk factors stratified by age [6]. The potential for proactive prevention relatively lower-burden individuals at younger ages is highlighted.

In the context of proactive prevention strategies, it is necessary to define what a “low-burden” or a “high-burden” population is. While we acknowledge that no absolute consensus will be achieved in terms of this distinction, we offer two illustrative examples of relatively “low-burden” populations. For instance, Fig 1 demonstrates that low- to mid-socio-demographic index populations currently have relatively lower mortality rates due to NCD risk factors, and therefore would benefit from proactive prevention efforts to maintain the lower NCD-related mortality rates [6]. Fig 2 portrays similar information, categorized by age rather than socio-demographic index, showing that younger individuals will largely benefit from proactive prevention as well [6]. We recognize and acknowledge that one of the limitations of a comparative risk assessment approach to estimating risk-attributable deaths or disability adjusted life years is that period effects are assumed for each age group when in reality present-day burden is a function of exposure in decades past. Proactive prevention therefore aims to decrease future length of exposure to NCD risk factors among these populations.

## Prioritizing proactive prevention: Impact and feasibility

**Fig 3:**
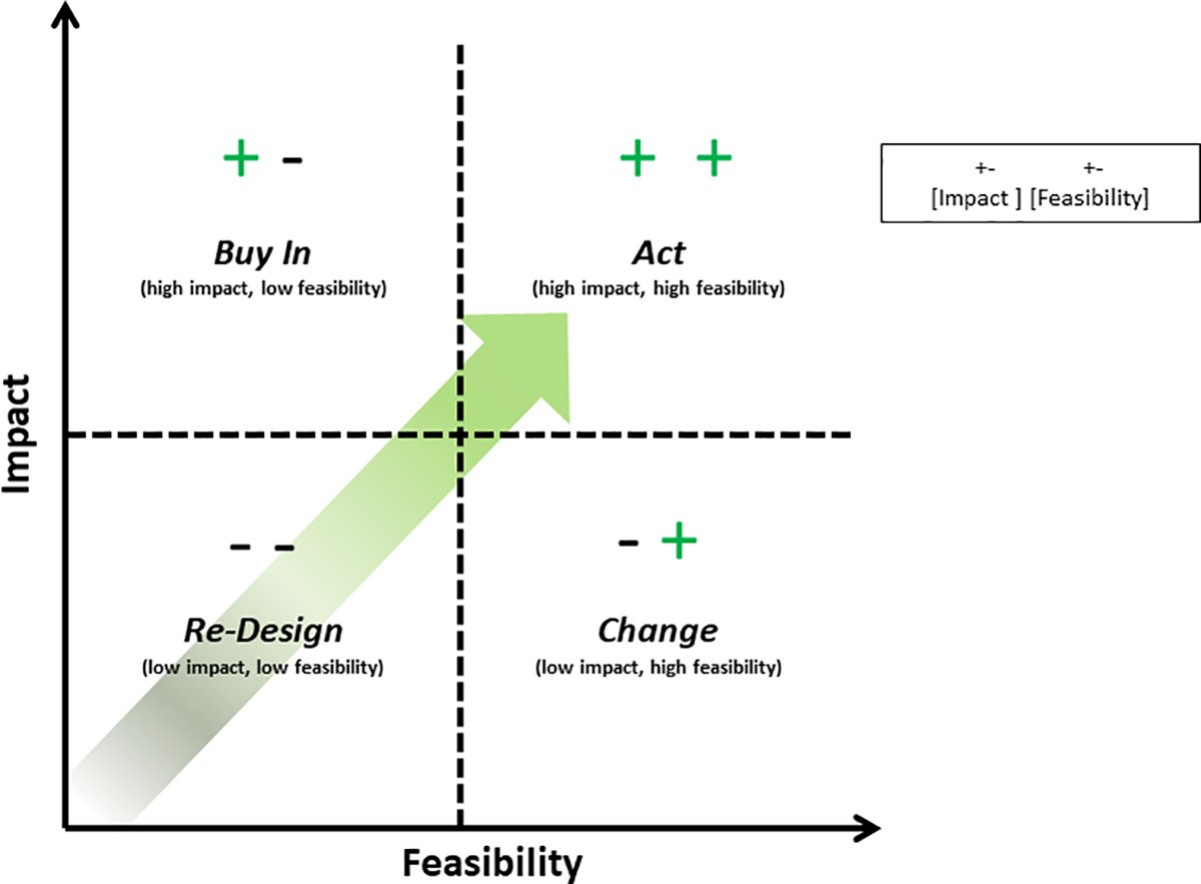
Impact and feasibility framework, illustrating the four graded categories of proactive prevention strategies: Act, buy in, change, re-design.

We propose a simple categorization scheme for potential proactive prevention interventions, “graded” along two dimensions: impact and feasibility. Each dimension—impact and feasibility—can be graded as “high” or “low” resulting in a simple 2x2 framework with four possible combinations: high-impact, high-feasibility (“**A**ct”); high-impact, low-feasibility (“**B**uy In”); low-impact, high feasibility (“**C**hange”); and low-impact, low-feasibility (“re-**D**esign). It should be noted, however, that these categories fall along a continuum (Fig 3), and there are no specific cut-offs to determine what is “high” vs “low” impact or feasibility. In addition, these assessments will likely vary based upon the specific target population or geographic region of implementation. We have intentionally made this framework simplistic as our hope is to promote improved utility for policymakers and program implementers to help prioritize strategic proactive prevention interventions.

When referring to “impact,” we incorporate the following elements: (a) *effectiveness* of the intervention on reducing disability and deaths, (b) *relevance to the intended recipients* taking into account current burden and future risk based on exposure over time, *(c)* long-term positive and negative *consequences* of the intervention as well as consequences of inaction, (d) *reach* (the number of persons served by an intervention, their representativeness, and equity), (e) *efficiency* in the intervention’s use of resources, (f) *sustainability* in terms of long-term inputs to maintain the intervention, (g) *scalability* which refers to the potential for the intervention to be scaled up to larger communities and populations, and (h) *expansion* potential to new populations or settings [7, 8].

Feasibility encompasses the (a) *acceptability* of the intervention to recipients and stakeholders, *demand* for the intervention, (b) *implementation* insofar as an intervention can be enacted as designed in a real-world setting, (c) *practicality* in terms of resource constraints, (d) *adaptability* to various situations, and (e) *integration* with regard to the necessary system change needed to incorporate an intervention into existing infrastructure and communities [9]. Finally, ethical and human rights considerations should underlie the entire process of considering potential interventions and determining their impact or feasibility.

In summary, proactive prevention efforts can be prioritized in relation to an intervention’s impact and feasibility. As illustrated in Fig 3, interventions in quadrant A (“Act”) should be prioritized for implementation. Quadrant B (“Buy In”) requires mobilization efforts to gain buy in from various stakeholders. Quadrant C (“Change”) are those which should be modified for improved effectiveness before implementation is attempted. Finally, quadrant D (“re-Design”) interventions should be re-designed until both improved impact and feasibility can be demonstrated. In the following sections, we describe examples of proactive prevention interventions that illustrate our proposed impact-feasibility framework. These examples are not meant to be exhaustive, but rather illustrative, as we recognize that there are many more interventions that can fit into each of the categories.

## High impact/high feasibility: Act

Interventions in the high impact/high feasibility category are the most vital and urgent to institute, they require us to **ACT**. These tend to be population-level interventions which are both effective and have a broad reach [3, 10]. This category usually involves government action in the form of fiscal policies or regulatory/legislative interventions and generally garner benefit by altering the choice architecture of citizens in favor of opting for the healthful intervention. They are often highly acceptable to the public with the public health benefit being understood and supported by the general population. These interventions sometimes have the additional beneficial consequence of being revenue-producing [11]. Exemplar interventions in this category are: excise taxes on tobacco, and trans-fatty acid elimination.

### High excise taxes on tobacco

The reduction of tobacco usage by a variety of policies has been extensively studied [12] and endorsed by the World Health Organization (WHO) [13]. Implementation of tobacco taxes are one of the most effective means of tobacco reduction when compared to other methods, and this intervention has been studied in many different countries using a variety of tax structures [14, 15]. In the short-term, it is second only to comprehensive smoke-free air laws, while in the long-term it has the largest overall effect [12]. Tobacco taxes also have the lowest estimated annual cost of tobacco control measures as estimated by the WHO, and have the added benefit of generating revenue that can be invested in health promotion efforts [12, 16].

### Trans-fatty acid elimination

Various methods to reduce the consumption of trans-fatty acids (TFAs) have been studied, including dietary counseling, food labeling, voluntary food reformulation, and legislation of TFA maximums [17]. The single intervention with the highest level of effectiveness in reducing daily dietary TFA intake has been legislation [17, 18]. As an upstream intervention, well implemented legislation has a population-level reach and impact that is substantial [19]. It is relevant population-wide as cardiovascular disease is a long-term consequence of TFA intake for all people. It is sustainable as high TFA foods are essentially eliminated as a dietary option. Despite the feasibility concerns such as acceptability and demand that accompany most public health legislative efforts, TFA bans have been in effect for many years in several places worldwide, with Denmark being an early adopter, as well as New York City [17, 18]. Other countries, such as South Africa and Columbia have mandatory limits, and the WHO is calling for a worldwide ban by the year 2023 [20].

## High impact/low feasibility: Buy In

High Impact/low feasibility opportunities should also be prioritized to slow the impending NCD crisis. These generally share some of the characteristics of the “Act” interventions above. These are usually far-reaching population-level interventions; however, they are often characterized by lower acceptability to governments due to high costs of implementation, long-term investment, practical constraints such as infrastructure changes, and market distortion. The potential impact of interventions in this category can be significant, but only if we can obtain **BUY IN** to overcome the initial barriers to these upstream interventions. The two examples illustrative of this category are: restructuring built environments to encourage physical activity and implementing subsidies for healthier fruits and vegetables.

### Restructuring built environments to encourage physical activity

Many studies have shown associations between built environment attributes and the amount of physical activity of people, with evidence accumulating on the health benefits of living in neighborhoods with well-connected street networks, pedestrian access, and recreational facilities [21–24]. Neighborhood features such as street connectivity, residential density, leisure space and availability of traffic devices improve neighborhood walkability and promote walking, leisure and transport related to physical activity such as biking. This has been demonstrated for young adults, the elderly and children [25–28]. For example, there is extensive evidence that the availability of parks, playgrounds, and green spaces as well as other neighborhood features such as walkability and safety, profoundly shape children’s daily physical activity patterns [29–33]. Moreover, a broader, more ambitious built environment agenda would integrate physical activity with road safety measures in urban settings and thus have large co-benefits with injury prevention. This is particularly relevant in low-income countries where rapid urbanization continues.

### Implementing subsidies for healthier fruits and vegetables

The WHO recommends that individuals consume at least 400 grams (equal to five servings) of fruits and vegetables (F&V) per day [34]. There are multiple barriers to achieving this recommendation. F&V and healthier diets generally cost more than foods of lower nutritional value [35]. There is a 22% supply gap in meeting current need for F&V, with low-income countries experiencing the largest gap [36]. There is substantial evidence of the benefit of subsidies for F&V on increased consumption. A review of studies employing subsidies to promote healthy food purchase and consumption (including F&V) showed that in 19 of the 20 reviewed studies, subsidies on healthier foods resulted in significant increase in the purchase and consumption of promoted products [37]. One large-scale example of F&V subsidization is the European School Fruit Scheme which since 2017 has been combined with a dairy scheme; the scheme provides free F&V to approximately 159,000 schools and is financed by a combination of EU, national, private, and parental contributions [37, 38]. In an evaluation of the scheme in Germany, an increase of F&V intake from 1.26 to 2.02 times per day was observed after one year’s delivery of the program [39]. In the United States, subsidization of F&V in the Supplemental Nutrition Assistance Program (SNAP) is associated with increased consumption of F&V [40, 41]. However, increasing the supply of F&V requires changing incentives within food production systems [42] and a shift away from the current emphasis on staple grain production [43]. Money budgeted toward ineffective subsidies, such as grain production or fossil fuels, have the potential to be reallocated toward F &V to overcome cost barriers [44]. Trade policies and the need to change dietary behavior also remain among the potential roadblocks, as there continue to be substantial challenges, both within countries and trans-nationally, to enact and implement the necessary policies to achieve this goal.

## Low impact/high feasibility: Change

Low impact/high feasibility interventions tend to be less “whole population”-based than the above two categories. They may only target certain segments of the population, reducing reach and overall impact, but are often easier to enact because aspects such as demand, practicality, and the ability to implement the intervention are far greater. This type of intervention less often needs governmental changes in policy or legislation, so there are fewer bureaucratic challenges. Public health interventions falling in this category would benefit from **CHANGE** to improve impact, as it is imperative to allocate available resources to those interventions with optimal public health benefit to curb the increases in NCDs. Examples of interventions that would benefit from change include: child obesity prevention strategies, and mass media campaigns to address NCD risk factors.

### Child obesity prevention strategies

Obesity in children and adolescents continues to increase worldwide and is recognized as a major contributing risk factor to premature mortality due to NCDs [45]. However, most school-based educational strategies have had little to no impact on prevention of obesity and have been characterized by sustainability challenges [4, 46, 47]. A recent modeling study assessed a package of interventions for NCD risk reduction among adolescents, targeting tobacco smoking, alcohol use, tax on sugar-sweetened beverages, and a school-based program for physical activity and healthy diet [4]. Although the study predicted a mortality benefit from the school-based program, its benefit-cost ratio was very low because costs were so high [4]. In summary, child obesity programs appear to need to be multi-faceted and include home- and community-based aspects. In addition, these programs must be supported by policy to address social, economic, and environmental determinants of health [46, 48, 49].

### Mass media campaigns to address NCD risk factors

Implementing mass media campaigns as public health initiatives has been common practice for decades [50]. Generally, mass media campaigns are a simple way to increase knowledge and awareness of an issue. However, while abundant, such campaigns are ultimately inconsistent in their impact. While some campaigns have shown moderate to strong evidence for benefit in combatting certain risk factors, such as tobacco use, physical activity, and nutrition, other research on alcohol use, certain cancer screenings, mental health, obesity and more have not shown evidence for benefit [51, 52]. Furthermore, impact is inconsistent and mixed across the literature for the same behaviors or conditions [53]. Certain changes that may improve impact could include: a) adopting strategies that have been successful by increasing the length of time and intensity, targeting specific audiences and cultural contexts, or restructuring the delivery (e.g. positive vs. negative messaging); or b) by expanding to a more comprehensive strategy, incorporating proven behavioral change theory, innovative information technology, as well as targeting policy and environmental factors, beyond solely providing public education and communication [51, 53, 54].

## Low impact/low feasibility: Re-Design

Public health interventions which fall in the low impact/low feasibility category should be **RE-DESIGNED**

While these interventions have the potential to have widespread impact, in practice they have generally had only modest impact on individual risk factor profiles and therefore are characterized by less overall impact. The effectiveness of the intervention itself may be questionable, or its cost high. Unlike the “act” and “buy in” options, these interventions are generally downstream and favor individual behavioral change, leaving individuals with unhealthy alternatives that may appear more desirable in the short-term. This limits acceptability and expansion potential. Two representative examples for this category are: altering food environments, and cookstove interventions to reduce household air pollution.

### Altering food environments

Redesigning grocery store aisles or lunch-rooms has been proposed as an intervention that leverages changing consumers’ choice architecture when they shop for food. It combines built environment strategies with socio-behavioral approaches to change and includes receding unhealthy foods to the background with healthier foods made more prominent. These interventions aim to consciously or sub-consciously increase preference for healthier options [55]. While theoretically attractive, there is limited evidence of low quality that redesigning food acquisition environments has substantial impact on food choices [55, 56]. The feasibility of grocery store re-design is limited by the fact that ‘junk’ food in general tends to be a leading source of income for grocery stores, therefore making a strategy likely to reduce revenue and profits less acceptable to store owners [57]. Second, in some settings, healthier food options tend to be less affordable than junk food (e.g. a bag of chips costing less than a fruit salad) [57]. Finally, the effect of redesigning food acquisition environments on actual food consumption and on overall health outcomes remains unknown.

### Cookstove interventions

Improved cookstove initiatives (ICIs) target reducing household air pollution (HAP) for individuals who utilize solid fuel sources such as firewood and charcoal for cooking in households or restaurant settings. Solid fuel is commonly used in LMICs [58], results in high levels of indoor air pollutants [59], and can lead to chronic respiratory diseases, CVD, and lung cancer [1]. ICIs aim to increase efficiency of energy use and reduce exposure to household air pollution [60]. The feasibility of ICIs is limited by partial adoption and intermittent use, reflecting persistence of cooking over open stoves [61]. In addition, the impact of ICIs on health outcomes is unclear. ICIs reduce HAP levels by 50–90%, although there is a lot of variability based on the type of stove used, with chimney stoves performing best [61, 62]. Despite this, the average post-intervention levels of HAP remain higher than those recommended by WHO, posing residual health risks to individuals [63, 64]. Recent systematic reviews reported inconclusive findings of ICIs on health outcomes [65]. Potential strategies to reduce household air pollution sufficient to exert health benefit include enabling complete adoption of ICIs [66], as well as encouraging the shift to cleaner fuel sources in addition to improved cookstoves [67].

## Discussion/way forward

The rationale to pursue an approach of proactive prevention is to expand the target for preventive interventions to include currently low-burden populations, in order to prevent future premature death and disability from NCDs and most efficiently use limited budget and healthcare resources. We have introduced a simple framework to evaluate potential proactive prevention interventions, assessing both impact and feasibility. As a result, it is possible to prioritize actions that are high-impact and high-feasibility, while working to address and change other interventions that are either less impactful or less feasible.

While there are already examples of proactive prevention that have been implemented, much more needs to be done to protect and support low-burden populations that are at potential future increased risk. We encourage policymakers, program planners, community leaders, and individuals to actively promote a proactive prevention agenda in their respective spheres of influence. Coupling policy interventions can maximize population health impact. Municipalities can be effective implementers of global and national goals and priorities, particularly if they subscribe to a “Health in All Policies” approach to decision making [68]. It will be important to maximize impact and feasibility of potential interventions and strategies. This will require transdisciplinary and multi-sectoral collaboration, as well as community engagement, to improve, re-design, change, and increase buy-in, in order to be able to act. We recognize that the pathway forward might not be straightforward, easy, or linear. However, it is important to act now, before it is too late.
